# Development of whole brain versus targeted dentate gyrus irradiation model to explain low to moderate doses of exposure effects in mice

**DOI:** 10.1038/s41598-018-35579-x

**Published:** 2018-11-22

**Authors:** M. Dos Santos, D. Kereselidze, C. Gloaguen, M. A. Benadjaoud, K. Tack, P. Lestaevel, C. Durand

**Affiliations:** 1 Institute for Radiological Protection and Nuclear Safety (IRSN), Research department of RAdiobiology and regenerative MEDicine (SERAMED), Laboratory of Radiobiology of Accidental exposures (LRAcc), Fontenay-aux-Roses, France; 2 Institute for Radiological Protection and Nuclear Safety (IRSN), Research department on the Biological and Health Effects of Ionizing Radiation (SESANE), Laboratory of experimental Radiotoxicology and Radiobiology (LRTOX), Fontenay aux Roses, France; 3 Institute for Radiological Protection and Nuclear Safety (IRSN), Research department of RAdiobiology and regenerative MEDicine (SERAMED), Fontenay-aux-Roses, France

## Abstract

Evaluation of the consequences of low to moderate doses of ionizing radiation (IR) remains a societal challenge, especially for children exposed to CT scans. Appropriate experimental models are needed to improve scientific understanding of how exposure of the postnatal brain to IR affects behavioral functions and their related pathophysiological mechanisms, considering brain complex functional organization. In the brain, the dorsal and ventral hippocampal dentate gyrus can be involved in distinct major behavioral functions. To study the long term behavioral effects of brain exposure at low to moderate doses of IR (doses range 0.25–1 Gy), we developed three new experimental models in 10-day-old mice: a model of brain irradiation and two targeted irradiation models of the dorsal and ventral dentate gyrus. We used the technological properties of the SARRP coupled with MR imaging. Our irradiation strategy has been twofold endorsed. The millimetric ballistic specificity of our models was first validated by measuring gamma-H2AX increase after irradiation. We then demonstrated higher anxiety/depressive-like behavior, preferentially mediate by the ventral part of the dentate gyrus, in mice after brain and ventral dentate gyrus IR exposure. This work provides new tools to enhance scientific understanding of how to protect children exposed to IR.

## Introduction

In France, in 2012, 20% of children under 5 years of age^1^ underwent computed tomography (CT), essentially for examination of the head and neck. The increasing use of this ionizing radiation (IR) medical procedure is linked to its considerable benefits for the diagnosis and medical follow-up of patients. Nevertheless, the long-term potential adverse effects of this practice, during brain ontogeny, notably on behavioral functions are unknown. Interest in evaluating the consequences of its use is therefore increasing in the medical and scientific community. The long-term consequences of low to moderate doses (less than around 1 Gy) of IR in humans have been suspected but are poorly described^2–4^. Because of the sensitivity of the childhood brain and of those patients’ long-life expectancy, we need to correctly evaluate the benefit/risk balance of those IR medical exposures^5^. Experimentally, behavioral disorders have been observed in adult rodents after 0.5 Gy whole-body irradiation^6–8^, though the direct effect of IR on the brain cannot be evaluated. Moreover, to understand the pathophysiology of radiation-induced behavioral disorders, the multi-scale anatomical and functional organization of the brain needs to be considered^9–11^. Brain functioning is indeed based on a wide range of plastic neural networks and neuronal interactions at the inter- and intra-structural levels. To shed light on the effects of low to moderate doses of IR, we need to assess the sensitivity of the brain and its different structures as their specific involvement in radiation-induced behavioral disorders.

Among brain structures, the hippocampus (HPC) in the medial temporal lobe of brain plays a major role in behavioral functions across species^12^. The HPC is composed of several sub-regions, which include the cornu ammonis and the dentate gyrus (DG). Adult neurogenesis, a process by which new mature neurons are continuously generated and functionally integrate into the existing neural circuitry, occurs throughout life in the DG^13–15^. This neuroplastic phenomenon is thought to regulate some hippocampal information processing. Interestingly, after exposure of the brain in pediatric patients to high doses of IR during radiotherapy, the temporal lobe appears to be critical in the appearance of neurobehavioral decline at adulthood, and altered neurogenesis is thought to be linked to this decline^16–18^. Moreover, the posterior/dorsal HPC is reported to be rather involved in learning and memory and the anterior/ventral HPC in behavioral response to stress and in modulating emotional and affective functions^12,19^. Neurogenesis in the adult DG seems to participate, in part, in this dorsal-ventral dichotomy of HPC functioning^15,20,21^. Thus, a new scientific challenge is to elucidate the involvement of alterations in the dorsal-ventral DG in behavioral disorders, following postnatal exposure of the brain at low to moderate doses of IR.

To address these questions, we developed an approach based on the use of three experimental models of IR (doses (≤1 Gy) in 10-day-old mice, an age that is critical in central nervous system development in terms of the onset of behavioral disorders in adulthood^7,22–24^. These three models are: (i) a model of head irradiation, (ii) a model of targeted irradiation of the dorsal DG, and (iii) a model of targeted irradiation of the ventral DG. Recent years have seen the emergence of new types of irradiation platforms that allow to mimic radiation therapy protocols. One such is the SARRP (Small Animal Radiation Research Platform), which delivers targeted radiation to preclinical animal models with high accuracy^25^. One board imaging system and dedicated treatment planning system enable multi-beam, multi-angle, and multi-plane irradiation. The technical characteristics of the SARRP appear to be appropriate for the design of specific treatment plans to tackle new scientific issues in the field of low to moderate doses of IR. Here we describe the technical procedure used to develop our experimental models. We then report the validation of our irradiation strategy, ballistically and functionally, by performing gamma-H2AX staining and tests of anxiety/depressive-like behavior, respectively.

## Material and Methods

### Animals

Pregnant female C57BL/6 mice were received from Charles River (L’Arbresle, France) between 14–17 days of gestation. They were housed alone with a nest in their cage, at an ambient temperature of 21 °C and under a 12/12 h constant light/dark cycle. Food and water were provided ad libitum. Females were checked for birth twice daily (08.00 and 18.00). Day of birth was designated day 0. Male offspring, randomly distributed on different groups, were submitted to the irradiation procedure at 10 days of age. After the procedure, they were euthanized or returned to their own cage, respectively, for immunohistochemical and behavioral tests. No cannibalism and rejection from mothers were observed when mice were back to their own cages. At 21 days of age, male offspring were put in groups of 5–6 individuals/cage for behavioral experiments. Experiments were performed in compliance with French laws and guidelines for animal experimentation (R.214–87 à R.214-126) and were approved by the IRSN Ethics Committee (D9203201).

### Irradiation platform and dose applied

The SARRP (Small Animal Radiation Research Platform, Xstrahl, Ltd., UK) is a precision radiation device composed of a common X-ray source attached to a gantry that can rotated between −180 and 180 degrees^25^. It can be used for irradiation and imaging purposes thanks to a flat panel detector available to acquire CBCT images. The reference conditions used for SARRP irradiation with MuriPlan software are a voltage of 220 kV and an intensity of 13 mA with inherent and additional filtrations of 0.8 mm of beryllium and 0.15 mm of copper, respectively (Supplementary Data I). The effective energy is about 69 keV and the half value layer is about 0.667 mm of copper. To achieve low to moderate doses of IR (0.25, 0.5, and 1 Gy), the dose rate in this condition is too high, which leads to a very short irradiation time. To increase irradiation time, an intensity of 3 mA was used to achieve a dose rate, in dose to water, at the isocenter of about 0.5 Gy/min at 1 cm depth in a water phantom.

### Magnetic resonance image acquisition

To achieve accurate targeting of the DG, the SARRP onboard CBCT guidance is insufficient to visualize brain structures. To visualize the DG, magnetic resonance (MR) images were acquired on a 4.7 Tesla preclinical MRI scanner (BioSpec 47/40 USR, Bruker, Ettlingen, Germany). Scan acquisition time was about 29 minutes per mouse with this sequence for 17 slices in 2D. MRI acquisition parameters are reported in Table 1.

**Table 1:** Acquisition parameters for MR images.

FOV (mm)	16 × 14
Acquisition matrix	256 × 222
Resolution (µm)	63 × 63
Cut thickness (µm)	350
Repetition time (ms)	4000
Echo Time (ms)	70

### Irradiation protocols

Ten-day-old mice were anesthetized using intraperitoneal injections of ketamine (40 mg/Kg)/xylazine (0.8 mg/Kg) solution and then positioned in a suitable support inside the SARRP. Three irradiation protocols were developed to irradiate: (i) brain, (ii) the dorsal DG and (iii) the ventral DG. Mice of the control group underwent scanner imaging like the other mice.

#### Ballistics

**Figure 1:**
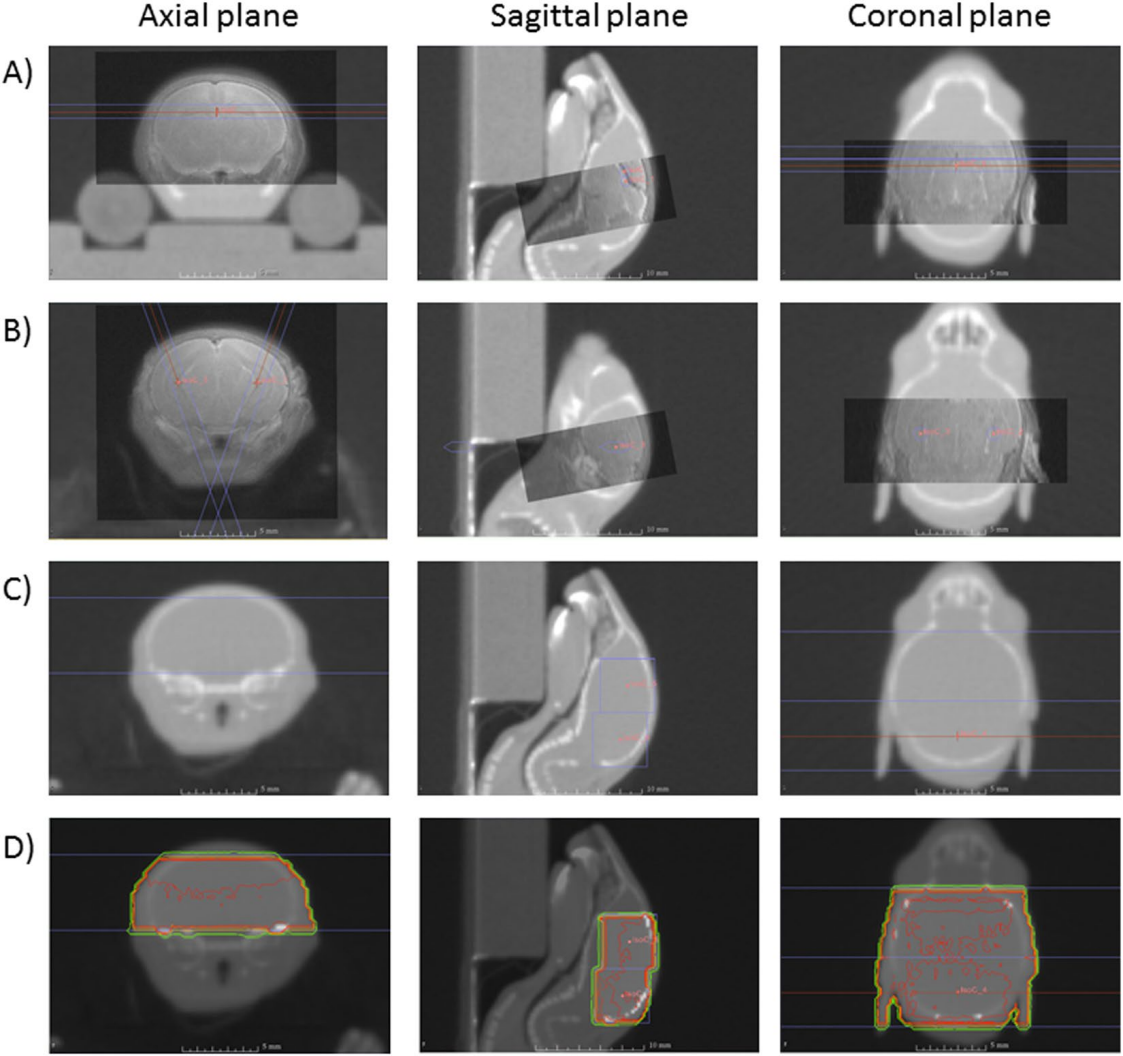
Illustration of the treatment plans used with the SARRP facility for irradiation of the dorsal DG (**A**) ventral DG (**B**) and brain (**C**). Panel D shows isodose lines for total brain irradiation.

To targeted irradiation, MR and CBCT images were recorded in parallel and manually superimposed on the SARRP treatment planning system called MuriPlan^26^. Concerning the dorsal DG irradiation, the strategy adopted was to position two isocenters one behind the other on the dorsal part of the DG with two beams at 180° to the sagittal plane with the circular 1 mm irradiation field (Fig. 1A). Using this treatment plan, we irradiate the dorsal DG and have a homogeneous distribution of the dose. For ventral DG irradiation, one isocenter was positioned in each ventral DG and beam irradiation was performed at ±20° to the sagittal plane with the circular 1 mm irradiation field (Fig. 1B).

For brain irradiation, parallel recording of images was not necessary. To irradiate most of the brain and to have a homogeneous distribution of the dose, we adopted the same strategy as that used for dorsal DG irradiation, namely two isocenters one behind the other with two beams at 180° to the sagittal plane with the 5 by 5 mm² irradiation field (Fig. 1C) and the corresponding isodoses (Fig. 1D).

**Figure 2:**
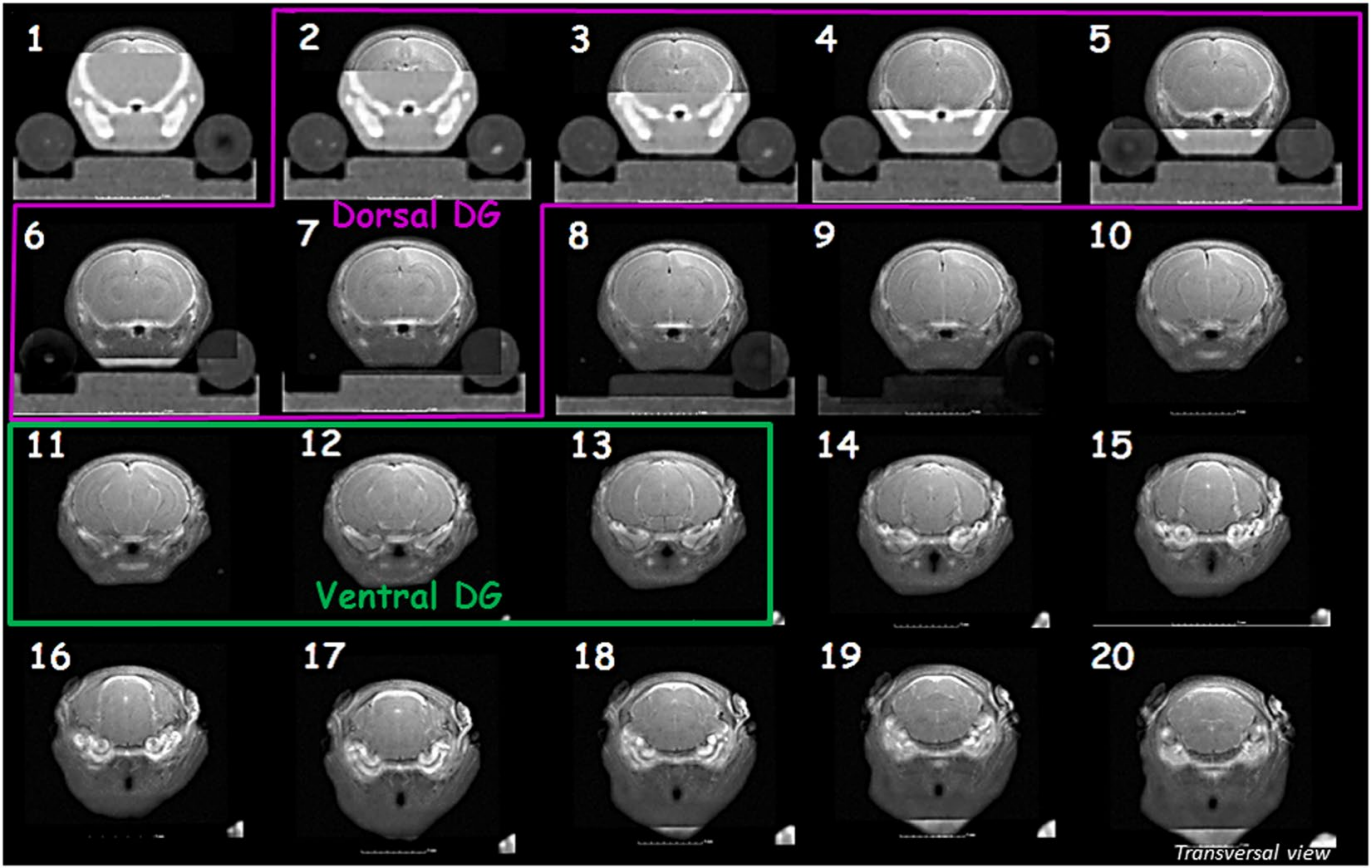
Co-registration images of transverse brain sections and delimitation of irradiation fields for the two targeted DG irradiation models. Dorsal and ventral DG transverse sections irradiated in those models are respectively framed in pink and green.

The different treatment protocols allow the irradiation of sections 2 to 7 and 11 to 13, respectively, in the dorsal DG and ventral DG irradiation models (Fig. 2).

### Beam ballistics specificity: validation

Ballistic validation in our models was performed using staining of gamma-H2AX, an indirect marker of DNA double stand breaks^27,28^.

#### Mouse brain tissue preparation

The gamma-H2AX response peaks 30 minutes to one hour after IR exposure^27,28^. Euthanasia of mice was performed one hour after irradiation. Mice were deeply anesthetized as described above and perfused intracardially with 10 mL of saline buffer and then with 10 mL of saline fixative solution (4% paraformaldehyde, pH = 7.4). Brain samples were excised and successively post-fixed for 12 hours in the same fixative solution before being cryoprotected in 30% sucrose/PBS 1X solution for 24 hours at 4 °C. Samples were then frozen in optimal cutting temperature compound at −45 °C and stored at −80 °C.

#### Mouse brain sections choice and immunohistochemistry procedure

**Figure 3:**
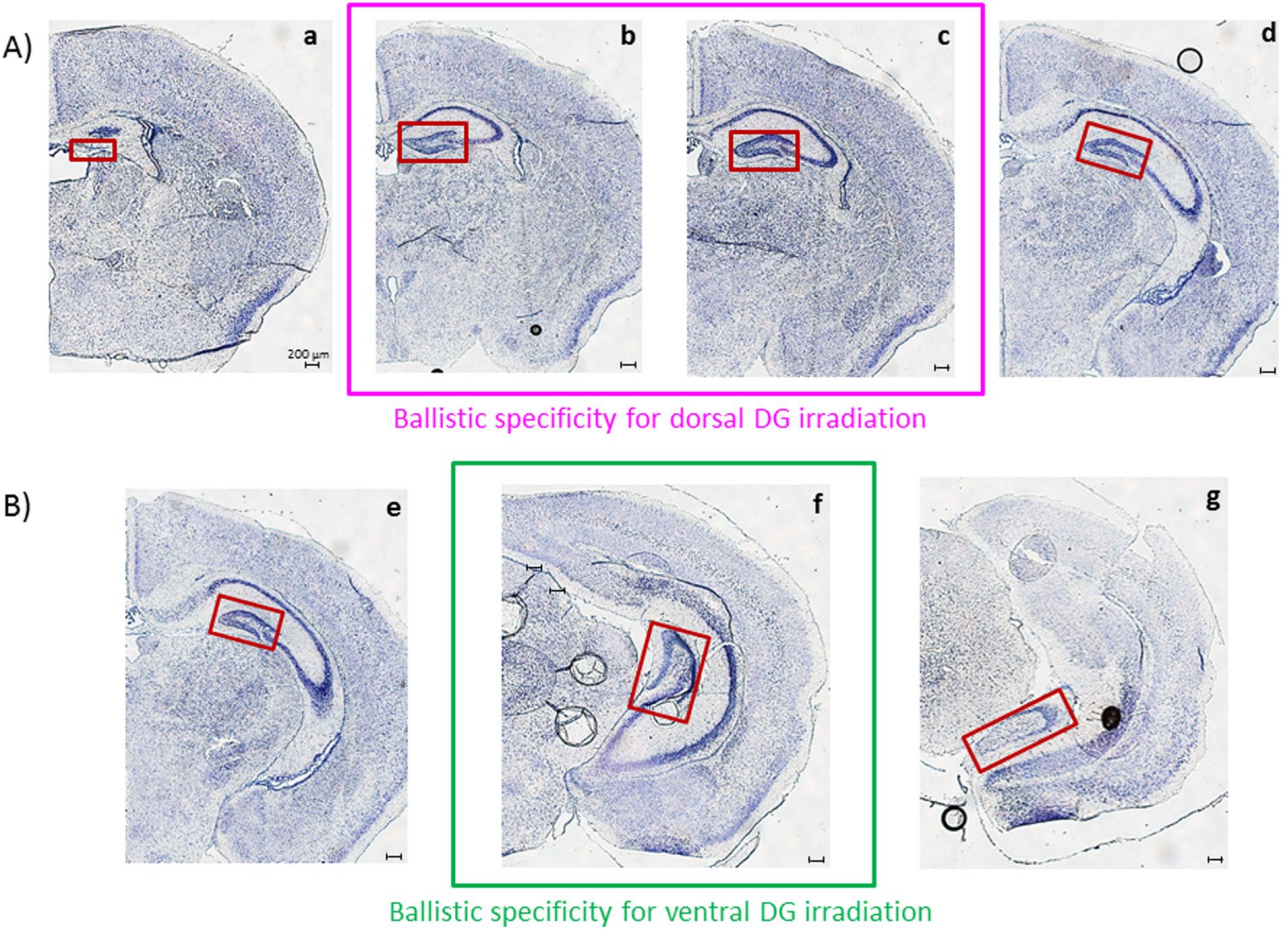
Distinct transverse sections observed for ballistic validation. Cresyl violet staining has been used here to reveal nervous tissue structures. The DG is framed in red in the photomicrographs. Four and three transverse sections were selected for the validation of dorsal (**A**) and ventral (**B**) DG model of irradiation, respectively. Sections a, d, e and f are supposed to be in the non-irradiated field, whereas sections b, c and f are supposed to be in the irradiated zones when following targeted irradiation protocols.

To validate our irradiation strategy and ballistic specificity in the two distinct targeted irradiation models, different brain sections were observed after gamma-H2AX staining in animals subject to targeted irradiation or brain irradiation and in control animals. For each configuration, the chosen sections are represented in Fig. 3. To validate the model of dorsal DG irradiation, the dorsal part of: control mice, brain irradiated mice and dorsal DG irradiated mice were analyzed (Fig. 3A,b,c). To validate the model of ventral DG irradiation, the ventral part of: control mice, brain irradiated mice and ventral DG irradiated mice were analyzed (Fig. 3B,f). From a technical point of view, transverse brain sections were 10 μm thick and we cut with a cryostat (Microm HMSSO) before being mounted on SuperFrost slides.

For each irradiation configuration, all sections were immuno-stained for all IR doses and for the control group. Tissue sections were treated successively for 30 min with 0.1% Triton X-100 in PBS 1X and with a protein block solution. They were incubated for one hour with an antibody diluent solution containing the primary rabbit monoclonal anti-gamma-H2AX (1/200; ab81299 Abcam Inc.). After 5-min washes with PBS 1X containing 0.2% Tween20, tissue sections were incubated with the same diluent solution containing the secondary donkey anti-rabbit IgG H&L, Alexa Fluor® 488 (1/5000; ab150061 Abcam Inc.). After the same washing procedure, slides were mounted with ProLong Gold Antifade Mountant with DAPI. These steps were performed at room temperature.

#### Microscopy and quantification

After immunostaining, observations were made for each configuration and for each irradiation dose. Brain sections of targeted irradiation models were compared with the similar ones of both the controls and the total brain (corresponding doses) irradiated animals using laser scanning confocal microscopy (LSM 780 NLO, Carl Zeiss MicroImaging).

Semi-quantitative ballistic validation was performed for both dorsal and ventral DG irradiation models by counting the number of nuclei expressing at least one gamma-H2AX foci in above 1000 cells, respectively in brain sections b and f (Fig. 3). This quantification was made in the targeted and brain irradiation groups and in the control group (n = 3–4 animals/group).

### Behavioral tests

Three anxiety/depressive-like tests (n = 10–22 animals for exposed groups and n = 33 for controls) were performed with the same animals during three consecutive days, 3 months after exposure. They were recorded by a video camera and were read by a trained observer unaware of the exposure conditions.

To have a sufficient number of mice per group, several days of irradiation were needed and several sessions of 3 consecutive days of behavioral tests were performed. For each irradiation day we have a control group. As there was no statistical difference between the results obtained with the different groups of control we can pool the results obtained during the distinct behavioral session and all the control animals were pooled in one group of control.

#### Elevated plus maze test

The elevated plus maze constituted of a wooden cross at a height of 50 cm with two open (30 cm × 5 cm) and two closed arms with walls (30 cm × 5 cm × 17 cm), arranged such that the arms of the same type were opposite each other and connected by a common open central platform (5 cm × 5 cm). Each animal was placed in the center of the maze, facing one of the open arms. The time spent in closed arms was recorded for 5 min, as an index of mouse anxiety. An arm entry was recorded if all four of the animal’s paws were in the arm^29^.

#### Marble burying test

It was done in Plexiglas cages (27 × 21 × 14 cm) containing 3 cm of fine sawdust. Twenty-five glass marbles (1 cm diameter) were evenly spaced on top of the sawdust. Mice were placed individually in the cages and left undisturbed for 30 min. Marbles were considered buried if two-thirds, or more, of their surface was covered by sawdust. The number of buried marbles is a measure of animal anxiety^30^.

#### Forced swimming test

It was conducted by gently lowering the mouse into a glass cylinder (height 17.5 cm, diameter 12.5 cm) containing 11.5 cm of water (23–25 °C). Test duration was 10 minutes. The mouse was considered immobile when it floated in the water, in an upright position, and made only small movements to keep its head above water. The duration of immobility, an index of stress response and depressive-like behavior, was quantified over the last 5 minutes of the test^31^.

### Statistical analysis

The results were compared between groups using mixed normal or Poisson regression models^32^ according to the nature of the parameter of interest: continuous response (elevated plus maze test and forced swimming test) or count data (Foci data and marble burying test scores), respectively.

In each model, the brain structures and radiation doses as well as their interactions were treated as “fixed” effects, while the experimental blocks were considered as random. The analysis was done using MATLAB Version: 8.2.0.701 (R2013b).

## Results

### The adopted irradiation strategy allowed the specific irradiation of either dorsal or ventral DG in ten-day-old mice

To validate the technical procedure put in place to irradiate millimetric brain structures, we performed gamma-H2AX staining. Gamma-H2AX is generated by phosphorylation of the histone H2A variant, H2AX, at serine 139, and is expressed in the HPC in 10-day-old mice in physiological conditions^33^. Nevertheless, its increasing expression is also observed after irradiation as a consequence of repair of DNA double-strand breaks^27,28^. We thus used it to validate the ballistic specificity of the models of irradiation developed.

Different brain sections were observed after gamma-H2AX staining in the two configurations of targeted irradiation (Fig. 3). In each case, those brain sections were compared with the similar ones of control animals and of total brain irradiation mice, at corresponding doses.

#### Dorsal and ventral DG irradiation configurations

**Figure 4:**
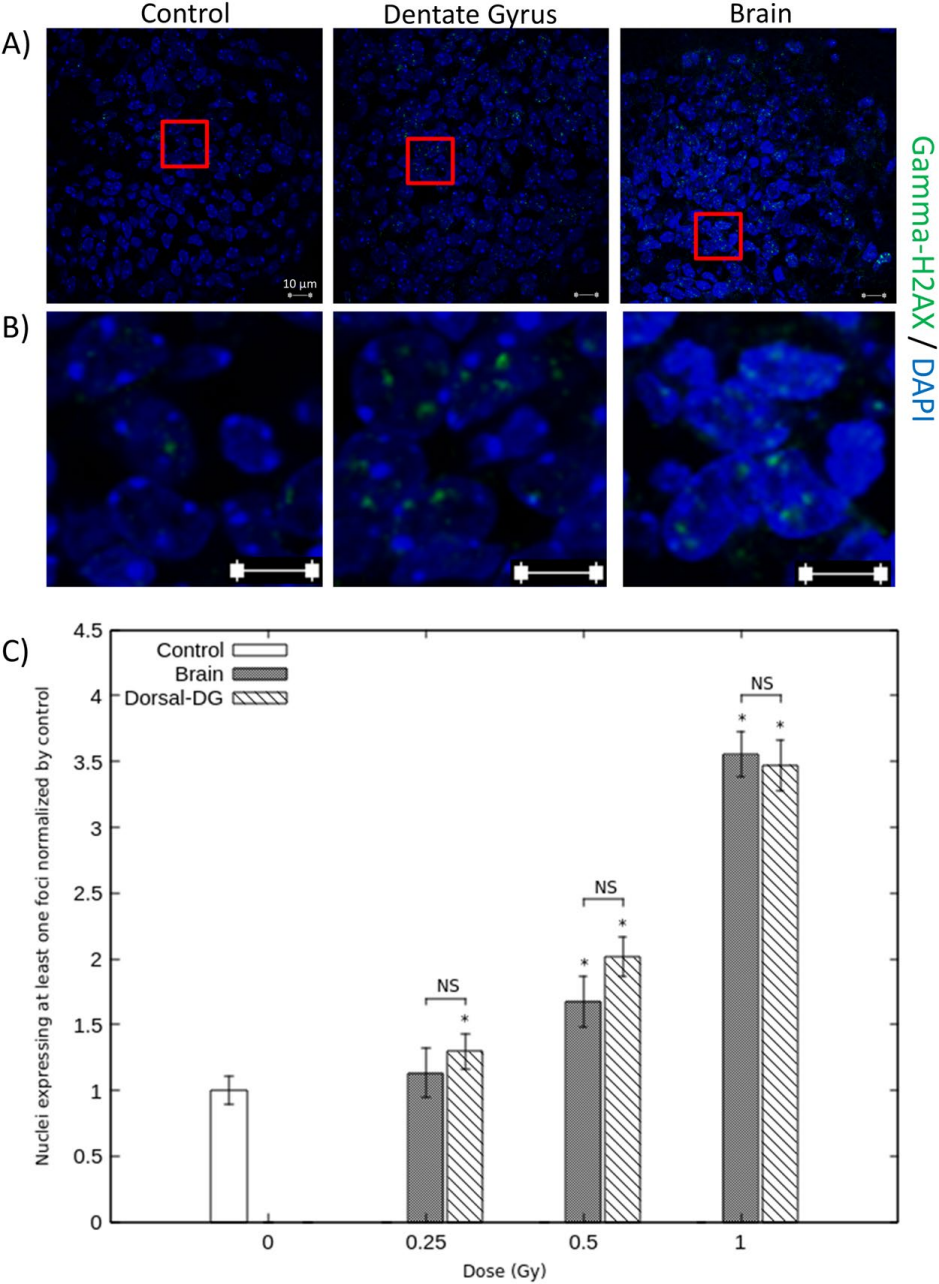
Ballistic validation for dorsal DG irradiation. Photomicrographs and quantifications were performed in brain section b (Fig. 3), which is supposed to be in the irradiated field following dorsal DG irradiation procedure. Panel (A) shows representative photographs of gamma-H2AX staining in DG cell nuclei of control mice and of mice 1Gy irradiated at dorsal DG and brain levels. Panel (B) shows enlargements of photographs represented in panel (A). Graph (**C**) represents the percentage of nuclei expressing at least one gamma-H2AX staining focus in controls, dorsal DG and brain irradiated animals, for each dose of IR. Data are represented as the mean ± sem of 3–4 animals per group and are normalized to their corresponding controls (*p < 0.05 *vs* controls).

**Figure 5:**
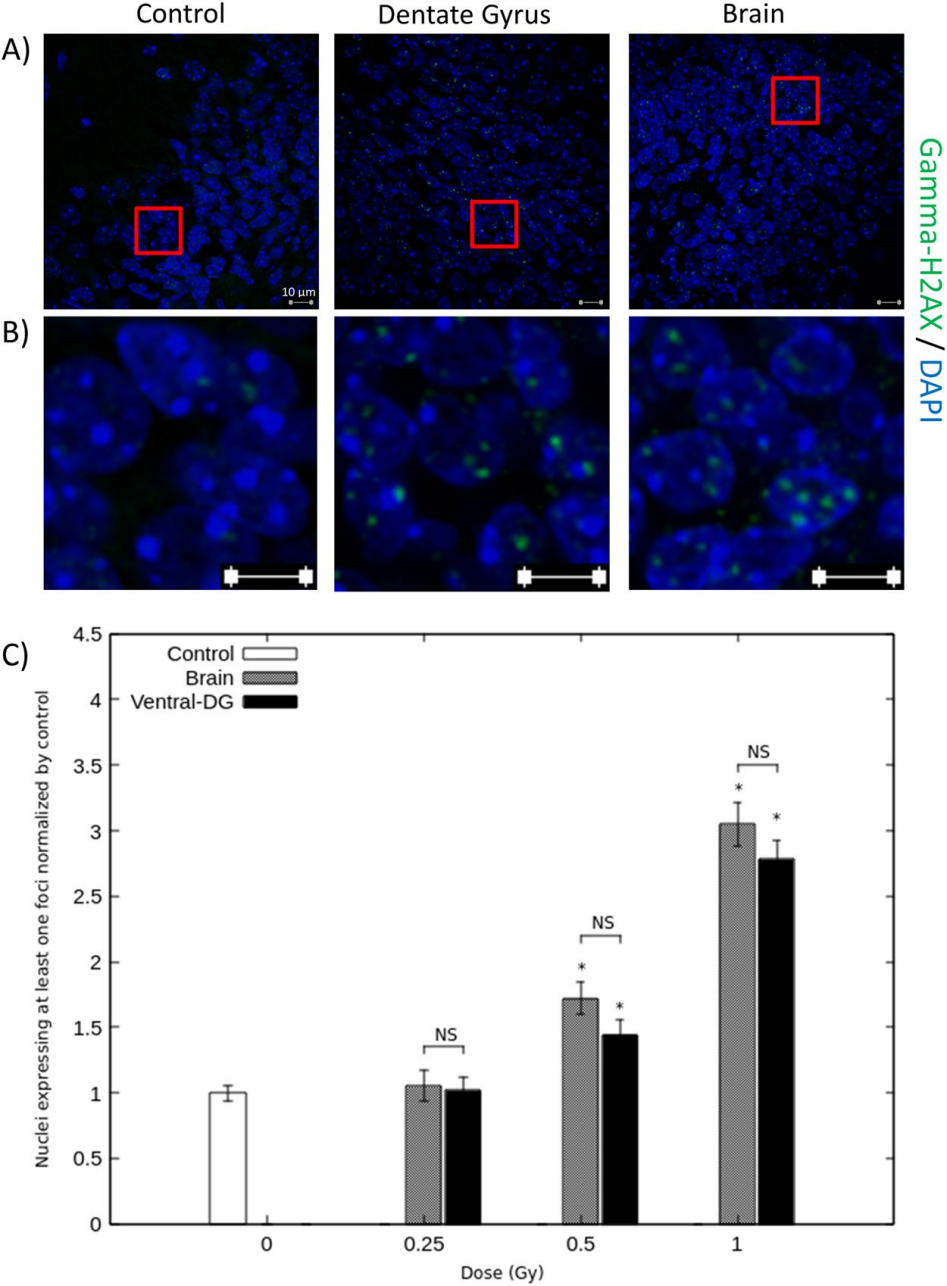
Ballistic validation for ventral DG irradiation. Photomicrographs and quantifications were performed in brain section f (Fig. 3), which is supposed to be in the irradiated field following ventral DG irradiation procedure. Panel (A) shows representative photographs of gamma-H2AX staining in DG cell nuclei of control mice and of mice 1Gy irradiated at the ventral DG and brain levels. Panel (B) shows enlargements of photographs represented in panel (A). Graph (**C**) represents the percentage of nuclei expressing at least one gamma-H2AX staining focus in controls, ventral DG and brain irradiated animals, for each dose of IR. Data are represented as the mean ± sem of 3 animals per group and are normalized to their corresponding controls (*p < 0.05 *vs* controls).

Figures 4 and 5 show the expression of gamma-H2AX staining considering, respectively, the dorsal and ventral DG irradiation configurations. Observations and quantifications were made in brain sections defined as located in the irradiated field after dorsal or ventral targeted irradiation, respectively, brain sections b and f (Fig. 3). The graphic C of Figs 4, 5 represents the percentage of nuclei expressing at least one gamma-H2AX staining, normalized to their corresponding controls. These results demonstrate a dose-dependent increase after targeted and brain irradiation in comparison with corresponding controls, for both irradiation configurations (Figs 4C and 5C). Considering dorsal ballistic validation, those increases are statistically different for doses above 0.25 Gy after dorsal targeted irradiation and above 0.5 Gy after brain irradiation (p < 0.05) (Fig. 4C). Considering ventral ballistic validation, those increases are statistically different from the dose of 0.5 Gy after brain irradiation and after targeted irradiation (Fig. 5C). Figures 4A,B and 5A,B show photomicrographs that illustrate this 3.5- and 3-fold increase in gamma-H2AX expression when 1 Gy was applied, after targeted and brain irradiations, respectively in dorsal and ventral DG sections. For each irradiation configuration and for each dose of irradiation, there were no significant differences between the targeted group and the brain irradiated group at corresponding doses (Figs 4C and 5C). Moreover, after targeted or brain irradiation, another parameter of irradiation appeared to be increased, in comparison with controls: the number of foci of gamma-H2AX staining per nucleus, as illustrated in Figs 4B, 5B.

Finally, observations made in non-irradiated fields after targeted irradiation confirmed the ballistic specificity of our models. For dorsal and ventral DG irradiation configurations, no difference in gamma-H2AX expression was observed in control animals and targeted irradiation models, respectively in sections a, d and e, g (Fig. 3), which are not supposed to be in the irradiated field. Moreover, comparing those brain sections with those of mice irradiated at brain level at corresponding doses, an increase in gamma-H2AX staining was observed after brain irradiation (data not shown).

Altogether those results validate our irradiation procedure and the ballistic specificity of our experimental models.

### The adopted irradiation strategy is functionally validated by anxiety and depressive-like behavior tests

**Figure 6:**
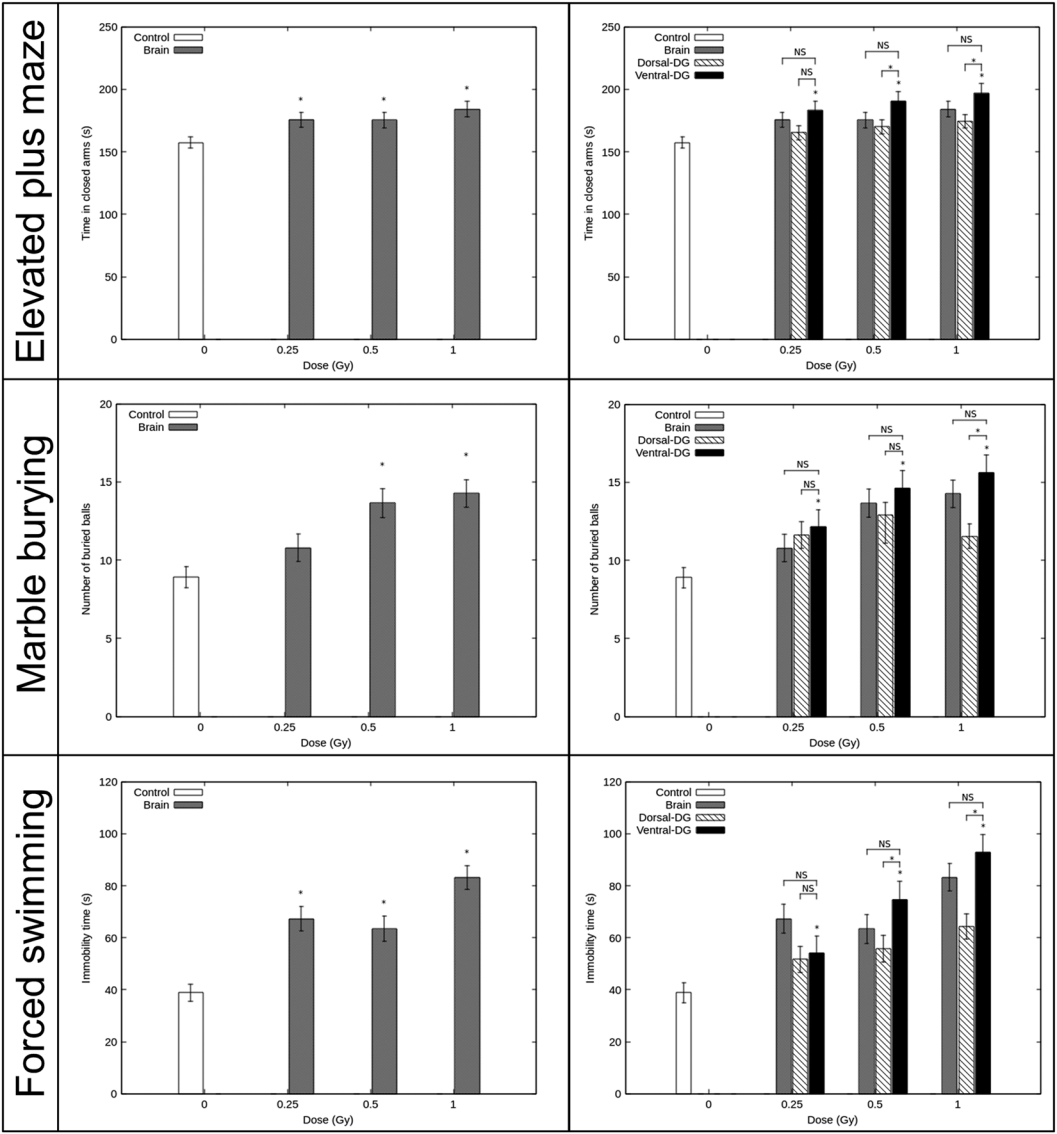
Functional validation of the experimental models. Results of anxiety/depressive-like behavior tests were obtained 3 months after irradiation in mice. The elevated plus maze test, the marble burying test and the forced swimming test (from top to bottom) were performed during three consecutive days. Results are expressed as mean ± sem of n = 10–22 animals for exposed groups and n = 33 for controls. For each behavioral test, there was no statistical difference between the control groups, therefore we pooled them (n = 33) and the different experiments. The left panel shows the effect of brain irradiation on behavioral functions in comparison with control mice (*p < 0.05 *vs* controls). In the right panel, behavioral functions in control mice, in brain irradiated mice and in dorsal DG irradiated mice in comparison with behavioral functions after ventral DG irradiation in mice is showed (*p < 0.05 *vs* ventral DG irradiated mice; NS is used to show non-significant difference *vs* ventral DG irradiated mice).

We used the distinct functioning roles of the dorsao-ventral HPC and performed anxiety/depressive-like behavioral tests to validate functionally the technical procedure used to irradiate millimetric brain structures. The results are shown in Fig. 6.

#### Elevated plus maze

The anxiety-like behavior of mice was assessed by the time spent in the closed arms in the elevated plus maze. Mice irradiated at level of the brain spent significantly more time in the closed arms than did the controls, from the dose of 0.25 Gy (p < 0.05, left panel). For the mice irradiated at the ventral DG, the time spent in the closed arms increased significantly at 0.25 Gy, 0.5 Gy and 1 Gy in comparison with the control group (p < 0.05, right panel). Moreover, for mice submitted to ventral DG irradiation, no significant differences were observed in the time spent in the closed arms compared with the brain irradiated animals at corresponding IR doses. Nevertheless, when comparing the results obtained for ventral and dorsal DG irradiated mice at 0.5 Gy and 1 Gy, a significant reduction of the time spent in the closed arms was observed after ventral DG-sparing irradiation (p < 0.05, right panel).

#### Marble burying test

To investigate further changes in anxiety-like behavior after irradiation, the mice were submitted to the marble burying test, since the willingness to bury marbles has often been interpreted as a sign of anxiety^30^. A significant increase was observed in interest in marble burying in the brain irradiated groups at 0.5 and 1 Gy, in comparison with the control group (p < 0.05, left panel).

For the mice irradiated at the ventral DG, the number of marbles buried was significantly increased from the IR dose of 0.25 Gy in comparison with the control group (p < 0.05, right panel). For each dose of exposure, no significant difference was observed between the ventral DG irradiated group and the brain irradiated group at corresponding doses. Nevertheless, mice irradiated at the ventral DG comparing with the ones irradiated at dorsal DG have significantly increased their buried glass marbles from the exposition dose of 0.5 Gy (p < 0.05, right panel).

#### Forced swimming test

The depressive-like behavior of mice and their response to stress were assessed by measuring the time they spent immobile during the 5 last minutes of the forced swimming test. For the brain irradiated group, the immobility time increased significantly when mice were irradiated at 0.25, 0.5 and 1 Gy compared with the control group (p < 0.05, left panel).

For the mice that underwent irradiation of the ventral DG, the immobility time increased significantly from the dose of 0.25 Gy, in comparison with control mice (p < 0.05, right panel). As in the two previous tests, no significant difference was observed between ventral DG irradiated mice and brain irradiated mice, at corresponding doses. When comparing the results obtained for ventral and dorsal DG irradiated mice, for the doses of 0.5 Gy and 1 Gy, the ventral DG-irradiated mice shown an immobility time significantly increased (p < 0.05, left panel).

## Discussion

We used SARRP facilities to develop an irradiation strategy dedicated to evaluating the effects of low to moderate doses of IR on the developing brain. Our results allow ballistic and functional validation of experimental models of irradiation that can be used in parallel to (i) evaluate brain radiosensitivity, (ii) evaluate the radiosensitivity of millimetric brain structures and to (iii) elucidate pathophysiological mechanisms involved in radiation-induced behavioral disorders, considering the functional brain’s organizational complexity.

The increase in gamma-H2AX staining observed after targeted irradiation did not differ statistically from the increase quantified after brain irradiation at the corresponding dose, for each configuration. As expected, this increase is dose-dependent and acts as an irradiation dose readout^34^. Altogether, the results obtained with the immuno-histochemical procedure validate the high accuracy and reproducibility of our irradiation strategy. Few facilities enable this type of irradiation. Conventional facilities, like X-ray cabinets or cesium/cobalt sources, have no onboard CT imaging system allowing brain visualization and do not permit accurate irradiation of such small fields and the localization of millimetric brain structures^21,35^. Conversely, the technological properties of SARRP facilities and the parallel recording of MR and CBCT images that favor visualization of structures of interest allowed this high millimetric ballistic specificity^36–41^. Until now, experimental studies with the SARRP have been performed to irradiate brain regions at high doses of IR to mimic radiotherapy protocols^38–40,42^ or to inhibit neurogenesis^37,43^. To tackle those goals, multiple beams, complex beam arrangements (multiple directions) and arc therapy have been used. Such strategies involve the delivery of moderate and high doses of IR to numerous brain structures around the targeted volume. The present work is to endorse technological and scientific choices in responding to new societal and scientific issues around the problematic of the use of CT-scan in children: evaluation of the behavioral consequences of brain exposure to IR doses ≤1 Gy and evaluation of the role played by specific brain structures in those disorders. In our case, focal irradiation design is thinking to improve our knowledge of how those IR impact some brain regions. In this context, our objective was to irradiate millimetric brain structures at low doses in a reliable way and to minimize the volume of other brain regions irradiated.

Technical and scientific choices were made to meet our goals. We first found reliable technical solutions to irradiate at low doses. By decreasing beam intensity and therefore dose rate, we were able to administer low doses of IR reliably and reproducibly, without modifying the X-ray energy spectrum by supplementary additional filtrations, as performed in other study^36^. Moreover, MR images use allowed the visualization of brain structure in contrary to the use of contrast agent intrathecally injected^37^ (Supplementary Data II). Intraperitoneal injections of ketamine/xylazine were used for mice anesthesia. Although, sensitivity to anesthesic is known for neonatal animals and isoflurane inhalation allow more rapid recovery, in the development of our irradiation strategy it was very difficult to use it. Indeed, a standard mask covers all the head of the animal and by removing the mask mice where not deeply sleeping leading to inaccuracies on ballistic specificity. Secondly, in a scientific point of view, we sought the best treatment plan strategy to develop irradiation models specific to one brain structure and that can be used in parallel. To irradiate the desired part of the DG and to avoid specific regions of the brain, the best compromise was chosen. Using our treatment plan strategy, we irradiated the dorsal or ventral DG, while avoiding other limbic structures, notably those implicated in emotional processes, and almost all the cortical brain (Fig. 2). Our treatment plans also avoided superposition of targeted irradiation of the dorsal and ventral parts of the DG (Fig. 2). This design allowed evaluation of the direct impact of IR on brain structures and its effects on behavioral functions. In the model of brain irradiation, critical structures as eyes, ears, and snout were avoided. So that, only the direct effect of IR on brain will be considered in our studies and so we could compare behavioral results obtained with the different models.

Anxiety/depressive-like behavioral tests performed demonstrated greater behavioral impairments after irradiation of the brain and of the ventral DG than after the dorsal one (Fig. 6, left and right panels). Moreover, for each dose of IR, comparison of impairments after irradiation of the ventral DG *versus* the whole brain and the dorsal DG, revealed, respectively, no statistically significant differences and statistically higher effects (Fig. 6, right panel, elevated plus maze test: 0.5 Gy; marble burying test: 1 Gy; Porsolt test: 0.5 Gy) after ventral DG irradiation. Altogether those results functionally validated our experimental irradiation models. Regardless of this validation, our experiments enhance understanding of the effect of low to moderate doses of IR on the brain and enable us to envision future studies using our three irradiation models.

Until now, even if some epidemiological studies suggest an increased risk for developing mental disorders in adulthood after postnatal exposure at low to moderate doses of IR^2,4^, their number is very few and their results are sometimes difficult to interpret. Our results add to understanding of the effects, on behavioral functions, of exposure of the developing brain to IR. To our knowledge, ours is the first experimental work that demonstrate anxiety and depressive-like impairments in adulthood after low to moderate IR exposure of the brain in 10-day-old mice (Fig. 6 left panel, elevated plus maze test: 0.25 Gy; marble burying test: 0.5 Gy; Porsolt test: 0.25 Gy). These results suggested an elevated emotional/affective state and an enhancement in the stress response. Those results differ from the ones obtain when brain exposure occurs in adulthood, as in the study of Son *et al*. in which no effect of IR was observed until the dose of 1 Gy using the tail suspension test^44^. Thus, the high radiosensitivity of the developing brain and the necessity to further investigations is confirmed. In those futures investigations, using targeted models will allow to assess the importance of the role played by the dorsal or ventral DG in those disorders and to elucidate the mechanisms by which IR affect the brain and its structures.

We have observed the direct sensitivity of the DG to IR during development and the consequences, at adult stage, of its exposure. Our results demonstrate more significant anxiety/depressive-like impairments after irradiation of the ventral DG than after the dorsal one. These findings are in accordance with the functional dissociation of the HPC along its longitudinal axis^12,19^. The functional role of the ventral HPC is notably linked to its preferential direct or indirect connections to brain structures involved in emotional/affective disorders such as the amygdala nuclei, the infralimbic, prelimbic and agranular insular cortices and the hypothalamus. Considering ventral DG irradiation, an indirect effect of IR on the brain regions mentioned above could explain the radiation-induced behavioral effects observed. Whether these effects, and notably the enhanced stress response measured using the Porsolt test, are mediated by alteration of neurogenesis in the adult DG needs to be investigated further. Alterations of ventral neurogenesis are supposed to be linked to enhanced responsiveness to stress^45,46^, through changes in the functioning of the hypothalamic-pituitary-adrenal axis. Nevertheless, this axis of stress can also be modulated without disturbance of neurogenic processes. Moreover, even if, to our knowledge, our models have the smallest field of irradiation, we cannot evade the fact that part of the hippocampal cornu ammonis is in the irradiated field. And, alterations in this region may also be involved in the behavioral impairments observed.

The results of our behavioral study had nevertheless to be put in to perspective with the potential effect of anesthetic injection processed during brain growth spurt. Ketamine injection in postnatal mice is indeed known to induce cell death and functional deficits in adulthood^47,48^ as isoflurane inhalation^49^. We can thus not evaluate the potential impact of postnatal animal anesthesia in our behavioral results. We used ketamine/xylasine injection to study anxiety and depressive like behaviors because we needed to have deeply anesthetized animals during irradiation and because Fredriksson and al in 2007 were not able to show any difference in the elevated plus maze test between animal injected with vehicle or ketamine in postnatal days. Nevertheless, even if we submitted all of our groups to the same procedure of anesthesia we need to take cautious interpreting our result regarding the difficulties to use anesthetic in mouse during brain ontogeny and because it has recently been demonstrated that irradiation can interact with ketamine^50^.

To finish, both the targeted irradiation models, in parallel with the irradiation brain model, could also be used to study radiation-induced cognitive impairments and their underlying mechanisms. Indeed, exposure of the brain to IR doses <2 Gy in very young children seems to lead to mental retardation and cognitive impairments in adulthood^2,3^, a phenomenon that should be studied in greater depth. Even though a dichotomic functional dissociation of HPC along its long axis can be made, this dissociation is not so simple. Notably, even if dorsal adult-born hippocampal neurons are sufficient to mediate some learning processes dependent on the HPC^15,20^, they need to work with the ventral ones in more challenging situations^21^. Our three models will be thus used to study radiation-induced cognitive disorders and the involvement of adult neurogenesis alterations of the dorsal and ventral DG hippocampus in their pathophysiology.

## Conclusion

The effects of low to moderate doses of IR on behavioral functions, because of brain exposure during diagnostic procedure, are not well established in humans and are a scientific issue of importance. To address this question we developed new experimental models on the SARRP. Our irradiation strategy was validated immunohistochemically and by behavioral studies. This work sheds light on scientific issues but could also leads to mechanistic studies to understand how IR impacts the brain. Using our models, it will be possible to study the direct/indirect effect of IR on brain structures, as well as bystander effects^51,52^, which are thought to mediate the biological effects of low-dose of IR at brain level.

## Electronic supplementary material

Dataset 1
