# Supplementary material for: Development of whole brain versus targeted dentate gyrus irradiation model to explain low to moderate doses of exposure effects in mice
Source: Sci Rep. 2018 Nov 22;8:17262. doi: 10.1038/s41598-018-35579-x (PMC6250717; doi:10.1038/s41598-018-35579-x)

Development of whole brain versus targeted dentate gyrus irradiation model to explain low to moderate doses of exposure effects in mice

M. Dos Santos^1^, D. Kereselidze^2^, C. Gloaguen^2^, M.A. Benadjaoud^3^, K. Tack^2^, P. Lestaevel^2^ and C. Durand^2,*^.

1. Institute for Radiological Protection and Nuclear Safety (IRSN), Research department in RAdiobiology and regenerative MEDicine (SERAMED), Laboratory of Radiobiology of Accidental exposures (LRAcc) Fontenay-aux-Roses, France.

2. Institute for Radiological Protection and Nuclear Safety (IRSN), Research department on the Biological and Health Effects of Ionizing Radiation (SESANE), Laboratory of experimental Radiotoxicology and Radiobiology (LRTOX), Fontenay aux roses, France.

3. Institute for Radiological Protection and Nuclear Safety (IRSN), Department of RAdiobiology and regenerative MEDicine (SERAMED), Fontenay-aux-Roses, France.

*Corresponding author: [christelle.durand@irsn.fr](mailto:christelle.durand@irsn.fr)

Supplementary data I: Energy spectrum of the SARPP.

The energy spectrum is calculated with SpeckCalc when a tension of 220 kV was employed with inherent and additional filtrations of 0.8 mm and 0.15 mm of Beryllium and Copper, respectively. This results in a spectrum with an effective energy of 69 keV.


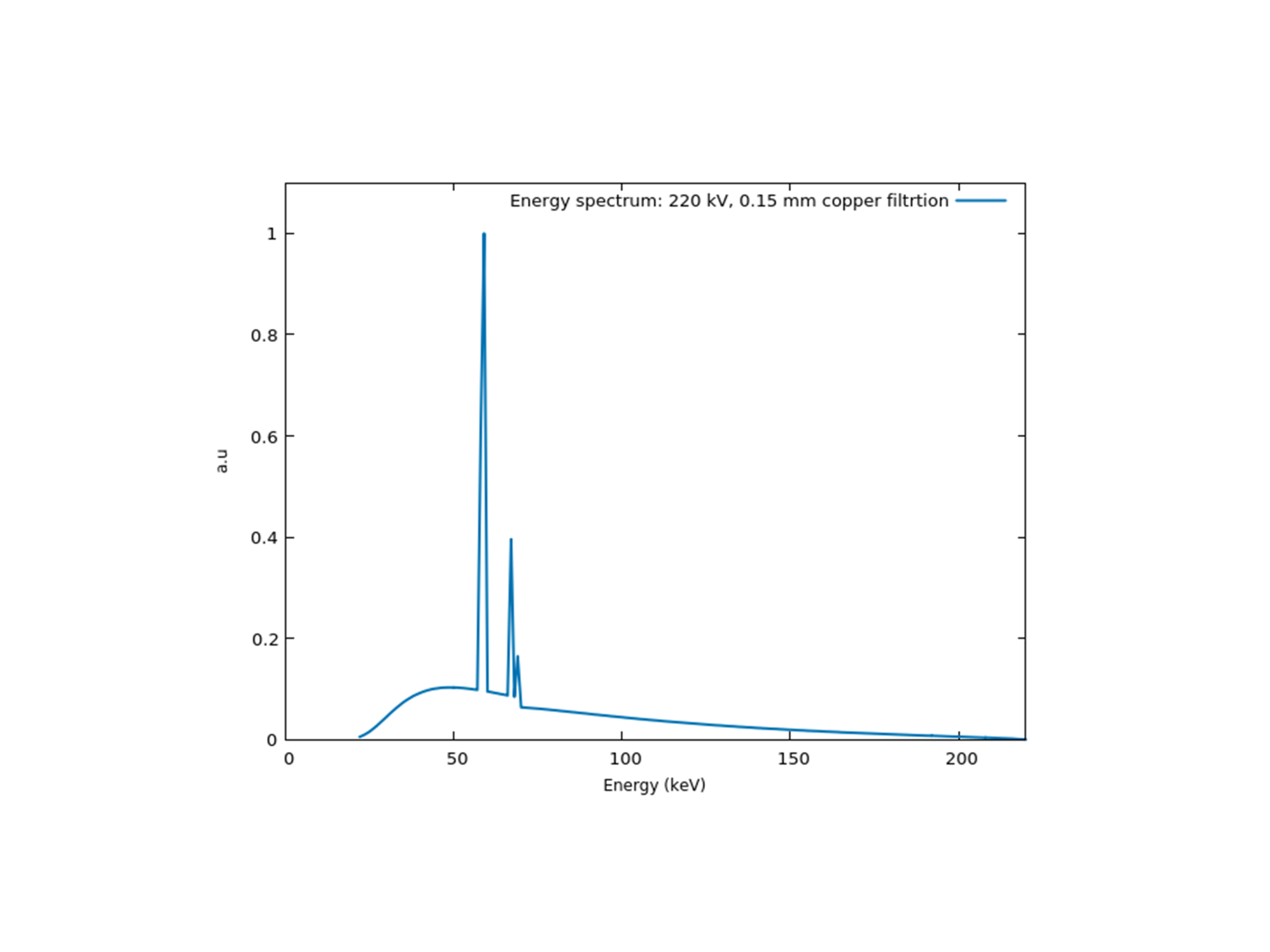


Supplementary data II: CBCT images obtained after the injection of a contrast agent.


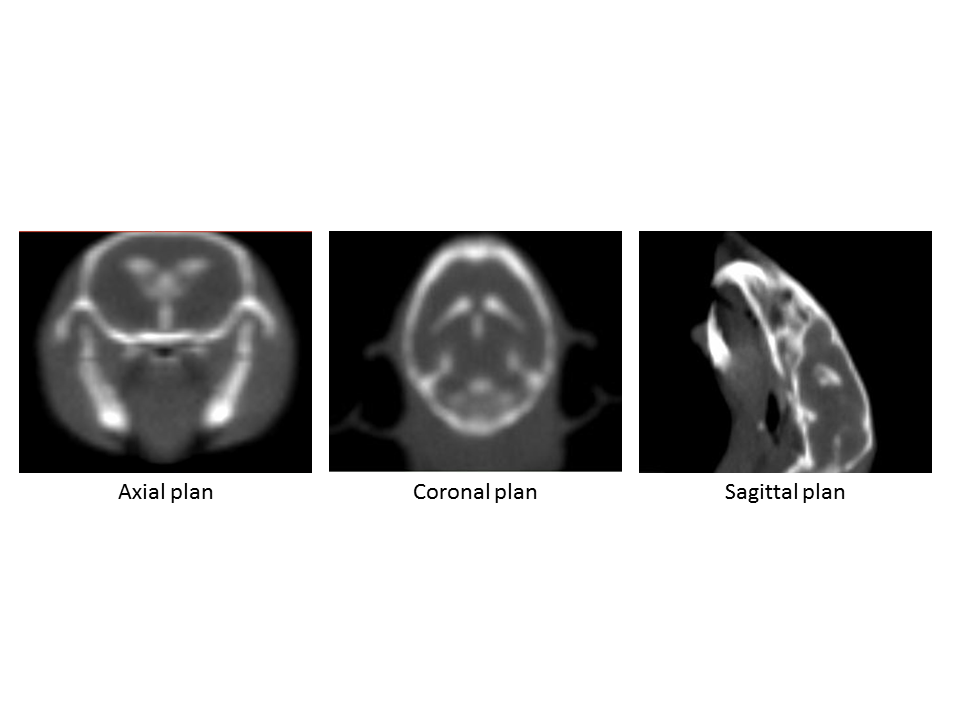

Supplement: Supplementary file 1 — Dataset 1 [file 41598_2018_35579_MOESM1_ESM.docx]
